# Structural, optical, opto-thermal and thermal properties of ZnS–PVA nanofluids synthesized through a radiolytic approach

**DOI:** 10.3762/bjnano.6.55

**Published:** 2015-02-23

**Authors:** Alireza Kharazmi, Nastaran Faraji, Roslina Mat Hussin, Elias Saion, W Mahmood Mat Yunus, Kasra Behzad

**Affiliations:** 1 Department of Physics, Faculty of Science, University Putra Malaysia, Serdang 43400, Selangor, Malaysia; 2 School of Chemistry, University of New South Wales, Sydney, NSW 2052, Australia; 3 School of Materials Science and Engineering, University of New South Wales, Sydney, NSW 2052, Australia; 4 Department of Physics, Science Faculty, Shahr-e-Qods Branch, Islamic Azad University, Tehran, Iran

**Keywords:** Fourier transform infrared spectroscopy (FTIR), specific heat, thermal conductivity, thermal effusivity, ZnS nanoparticles

## Abstract

This work describes a fast, clean and low-cost approach to synthesize ZnS–PVA nanofluids consisting of ZnS nanoparticles homogeneously distributed in a PVA solution. The ZnS nanoparticles were formed by the electrostatic force between zinc and sulfur ions induced by gamma irradiation at a dose range from 10 to 50 kGy. Several experimental characterizations were conducted to investigate the physical and chemical properties of the samples. Fourier transform infrared spectroscopy (FTIR) was used to determine the chemical structure and bonding conditions of the final products, transmission electron microscopy (TEM) for determining the shape morphology and average particle size, powder X-ray diffraction (XRD) for confirming the formation and crystalline structure of ZnS nanoparticles, UV–visible spectroscopy for measuring the electronic absorption characteristics, transient hot wire (THW) and photoacoustic measurements for measuring the thermal conductivity and thermal effusivity of the samples, from which, for the first time, the values of specific heat and thermal diffusivity of the samples were then calculated.

## Introduction

Over the past decade, the unique properties of nanometer-scale semiconductors have been investigated in the field of synthesis and characterization. Among the II–VI compounds, ZnS as a direct wide band-gap semiconductor (*E*_g_ ≈ 3.6 eV) [1], has gained more attention due to its potential applications in optoelectronics [2], lasers [3] and solar cells [4].

During the fabrication of devices that utilize semiconductor nanoparticles (NPs), such as ZnS NPs, the tendency of particles to agglomerate needs to be taken into consideration. The use of organic polymers as a host can help to prevent agglomeration and mobilization of the semiconductor nanoparticles [5]. Moreover, the incorporation of inorganic and organic materials enhances and combines the properties of both phases in the final product [6]. Poly(vinyl alcohol) (PVA) is one of the most suitable polymers [7] and has been reported in numerous publications to be a good choice for preparing colloidal suspensions due to its significant advantageous such as processability and high transmittance [8]. Various physical and chemical routes have been used to synthesize these nanocomposites such as microwave irradiation [9], chemical synthesis [10], sputtering [11], sol–gel [12], solid state synthesis [13] and gamma irradiation [14]. Among these methods, gamma irradiation has been considerably employed in various works because it can be applied under ambient conditions and at room temperature. In addition, this method provides size control and avoids impurities originating from chemical initiators [6].

The interest in measuring the thermal conductivity (*k*) and the thermal effusivity (*e*) of nanofluids containing semiconductors has increased [15] because of their increasing use in devices [16]. The photoacoustic (PA) effect has been demonstrated to be a valid technique for these measurements. It has the advantage of being a non-radiative de-excitation process following the absorption of light [17–18]. The aforementioned methods consequently provide the possibility for calculating specific heat (*C*_p_) and thermal diffusivity (α).

In this work, structural, optical, opto-thermal and thermal properties of ZnS–PVA nanofluids synthesized through a room temperature radiolytic method were studied, since these properties are considerable factors in optoelectronic devices. To the best of our knowledge, this is the first report on thermal characteristics of ZnS–PVA nanofluids in which thermal diffusivity and specific heat of nanofluids were calculated by using formerly known values of thermal conductivity and effusivity, which were acquired by using transient hot wire (THW) and photoacoustic methods.

## Results and Discussion

According to [19], the interaction of gamma radiation with the aqueous solution generates aqueous electrons (e^−^_free_) that reduce S_2_O_3_^2−^ to S^2−^. Zinc ions (Zn^2+^) are already present in the solution and through the combination of sulfur and zinc ions ZnS nuclei are formed, which agglomerate into ZnS NPs. The mechanism of this reaction is as follows:

[1]
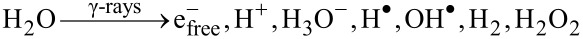


[2]
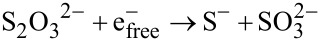


Thiosulfate ions (S_2_O_3_^2−^) were obtained by dissolving sodium thiosulfate (NaS_2_O_3_) in the PVA solution.

[3]
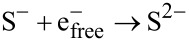


[4]
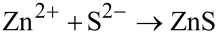


[5]
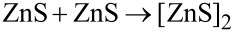


[6]
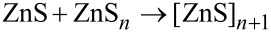


Equation 5 and Equation 6 indicate the agglomeration of ZnS NPs. The PVA matrix limits this agglomeration and leads to a smaller NP size.

The first noticeable hint to the formation of ZnS is the change of color of the samples from transparent to white. It was also observed that the viscosity of the final product was increased by increasing the dose from 10 to 50 kGy. This increment is due to the residual amount of acetate, which will be discussed later.

**Figure 1:**
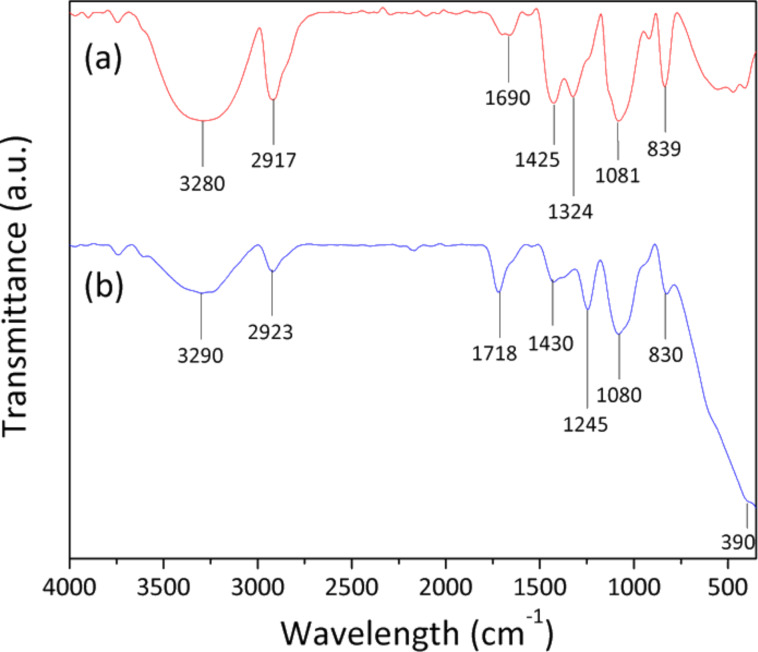
FTIR spectra of (a) PVA (b) PVA–ZnS.

Figure 1 depicts the FTIR spectra of PVA and the PVA–ZnS nanofluid. The main peaks of PVA were observed at 3280, 2917, 1690, 1425, 1324, 1081 and 839 cm^−1^. These peaks are assigned to the O–H stretching vibration of the hydroxy group, CH_2_ asymmetric stretching vibration, C=O carbonyl stretch, C–H bending vibration of CH_2_, C–H deformation vibration, C–O stretching of acetyl groups and C-C stretching vibration, accordingly [20–22]. Similar peaks were observed for the ZnS–PVA nanofluid in addition to two new peaks located at 1245 and 390 cm^−1^ due to the C–H wagging and ZnS NPs, respectively [22–23]. These results have been sorted in Table 1.

**Table 1:** The FTIR results of PVA and ZnS–PVA nanofluid.

assignment	observed wavelength for PVA (cm^−1^)	observed wavelength for ZnS–PVA (cm^−1^)	reference

OH stretching	3280	3290	[20–21]
CH_2_ asymmetric stretching	2917	2923	[20–21]
C=O carbonyl stretching	1690	1718	[20–22]
CH_2_ bending	1425	1430	[20–21]
C–H deformation	1324	—	[20–21]
C–H wagging	—	1245	[22–23]
C–O stretching	1081	1080	[20–21]
C–C stretching	839	830	[20–21]
ZnS NPs	—	390	[23]

**Figure 2:**
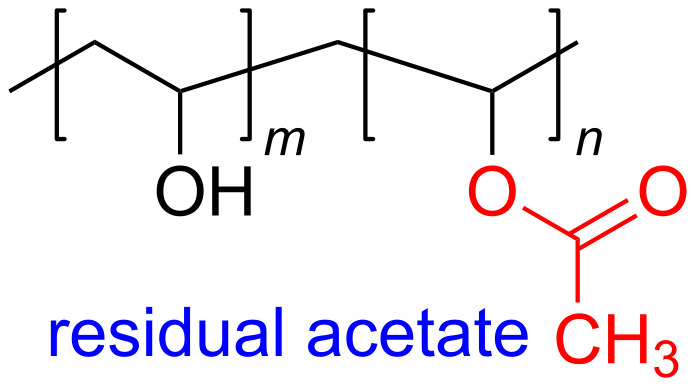
Illustration of hydrolyzed PVA.

According to [24], residual acetate can acetylate PVA, as illustrated in Figure 2. Indeed, a copolymer of vinyl alcohol and acetate is produced in a ratio of *m* and *n* mol %, in which *n* is significantly smaller than *m* due to the low concentration of residual acetate. Despite the low amount of acetate, the weight of the polymer chain is increased to some extent as a consequence of this process that results in poly(vinyl alcohol-*co*-vinyl acetate). Therefore, it can be envisaged that the viscosity is increased as well.

Generally, the vibrational spectra of PVA exhibits the characteristics of the vinyl alcohol monomer and it is expected to reveal 17 modes of vibrations [22]. The C–H wagging mode is expected to appear at about 1240 cm^−1^ for PVA. In this experiment, the C–H wagging mode appeared only after the acetylation process, which can be explained by a structure deformation of the PVA backbone. Another expected vibrational mode for PVA is the vibration mode of carbonyl stretching at about 1700 cm^−1^. The carbonyl stretching vibration was observed at 1690 cm^−1^ and 1718 cm^−1^ for PVA and ZnS–PVA nanofluid, respectively. The intensity of the carbonyl stretching mode was increased in the ZnS–PVA nanofluid, which suggests the presence of more carbonyl sites. The reason for this is the interaction of gamma radiation with the aqueous solution that caused the breaking of –H and –OH bonds, and therefore the formation of more carbonyl double bonds (C=O) [20]. Consequently, the intensity of peak corresponding to the carbonyl stretching vibration was increased and shifted toward higher wavelengths from 1690 to 1718 cm^−1^. Despite the acetylation of PVA moieties, the chain structure of PVA is, in general, breaking upon increasing the dose due to the bond scission because of the high energy of irradiation [20].

**Figure 3:**
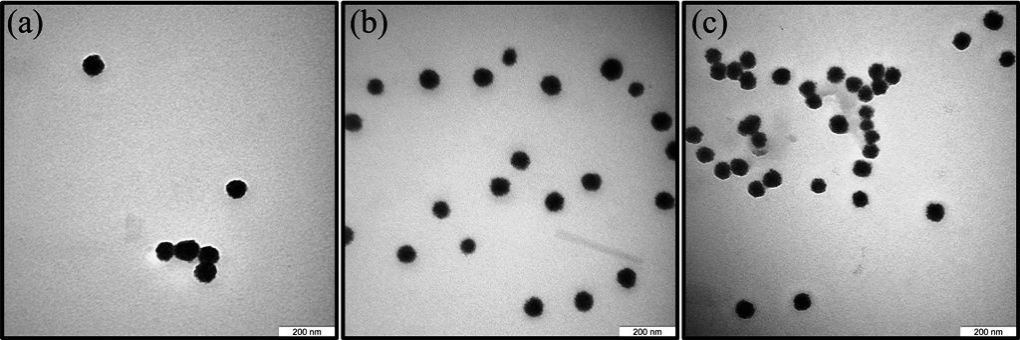
TEM images of ZnS NPs within PVA matrix at (a) 10 kGy, (b) 30 kGy and (c) 50 kGy dose.

Figure 3 shows the TEM images of ZnS NPs after irradiation with doses of 10, 30 and 50 kGy, respectively. The ZnS NPs are spherical and exhibit a homogeneous distribution, especially at higher radiation doses. The average sizes of the ZnS NPS were found to be 53, 57 and 59 nm for samples irradiated with doses of 10, 30 and 50 kGy, respectively. An increase of particle size with increasing dose in this study indicated that when more metal ions present in the sample the increased number of sulfur ions induced by gamma radiation agglomerated more ZnS units to enlarge the particle size at higher doses. Moreover, the number of ZnS NPs was increased when increasing the dose from 10 to 50 kGy because more ZnS NPs were formed due to an increasing number of non-metal ions in the solution. This increment in number supports the enhancement of some related phenomena such as intensity of absorption which will be later discussed.

**Figure 4:**
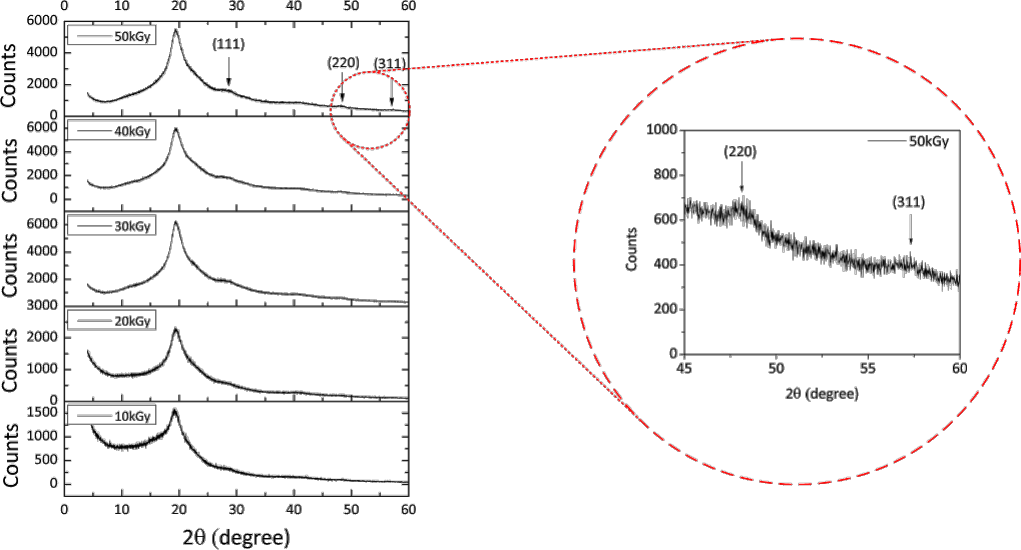
XRD pattern of ZnS NPs mediated by PVA from 10 to 50 kGy doses.

Figure 4 shows the XRD pattern of ZnS–PVA nanofluid samples synthesized with 10 to 50 kGy doses showing the structural characteristic and formation of ZnS NPs. The main observed peak at 19.5° is related to PVA in the solution [25]. The observed peaks at 2θ values of 28.13°, 47.69° and 57.42° were assigned to the (111), (220) and (311) lattice plane spacings of cubic ZnS, (zinc blende, ICDD PDF 00-065-0309) with a lattice parameter of 5.4 Å and 157.46 Å^3^ cell volume.

**Figure 5:**
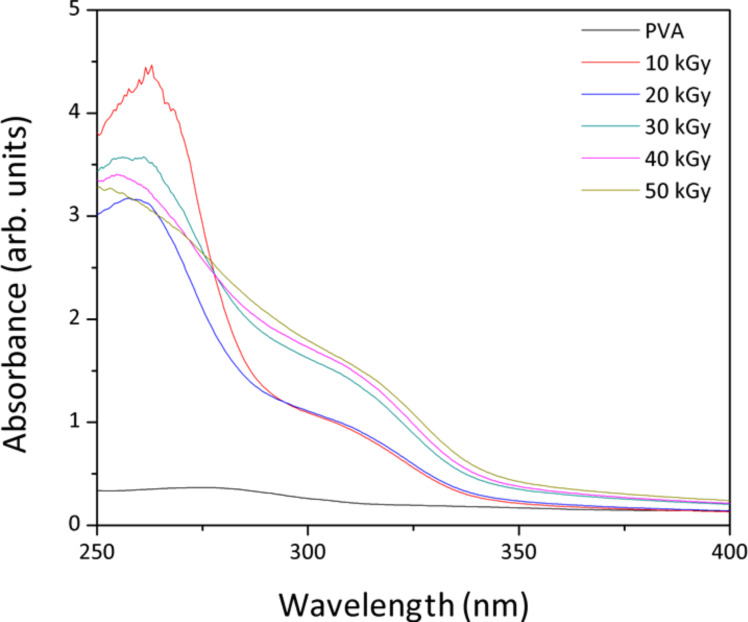
Optical spectra of ZnS–PVA nanofluids synthesized at various doses.

The absorption spectra of ZnS–PVA nanofluid samples are depicted in Figure 5. The absorption in the wavelength range of 310 to 320 nm varies for irradiated samples from 10 to 50 kGy doses. Consequently, a slight blue shift occurred compared to the absorption maximum (340 nm) of bulk ZnS [26]. Additionally, the result indicated an enhancement in the intensity of the absorption spectra for irradiated samples at higher doses due to the higher number of ZnS NPs. The peak located at 260 nm is due to the formation of some chromophores as a consequence of carbonyl formation in the PVA structure upon irradiation [20]. The intensity of this peak increases, which indicates an increase of the carbonyl concentration in agreement with the FTIR results.

**Figure 6:**
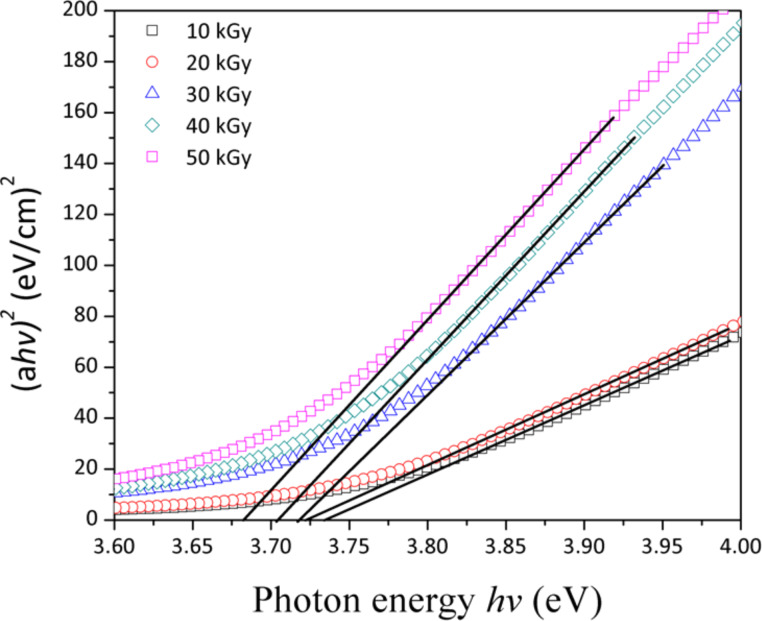
Optical band gap energy of ZnS NPs after irradiation with doses of 10 to 50 kGy.

The optical band gap energy of the ZnS NPs was estimated through the Tauc equation as follows [6]:

[7]
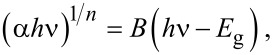


where α is the absorption coefficient, *h*ν is the photon energy of the incident light, *E*_g_ is the band gap energy, *B* is a constant and *n* depends on the type of transition and is 1/2, 1, 3/2 and 2 for allowed direct, allowed indirect, forbidden direct and forbidden indirect, respectively. The absorption band gap of ZnS NPs was estimated by extrapolating the linear portion of (α*h*ν)^2^ as a function of *h*ν. Consequently, the intercept of the extrapolated line with the x-axis, indicates the band gap energy of the ZnS NPs, as shown in Figure 6.

The absorption coefficient (α) was calculated by α = 2.303 *A*/*d* where *A* is the absorbance and *d* is the thickness of the sample [27]. The band gap of ZnS NPs decreased from 3.74 to 3.68 eV with increasing dose from 10 to 50 kGy due to increasing particles size; however, these values are generally higher than the band gap energy of bulk ZnS 3.6 eV [1]. The band gap energy implies a very weak quantum confinement effect. Since the wave functions of electrons and holes are still overlapped, the movement of the excitons instigates a weak confinement [28].

**Figure 7:**
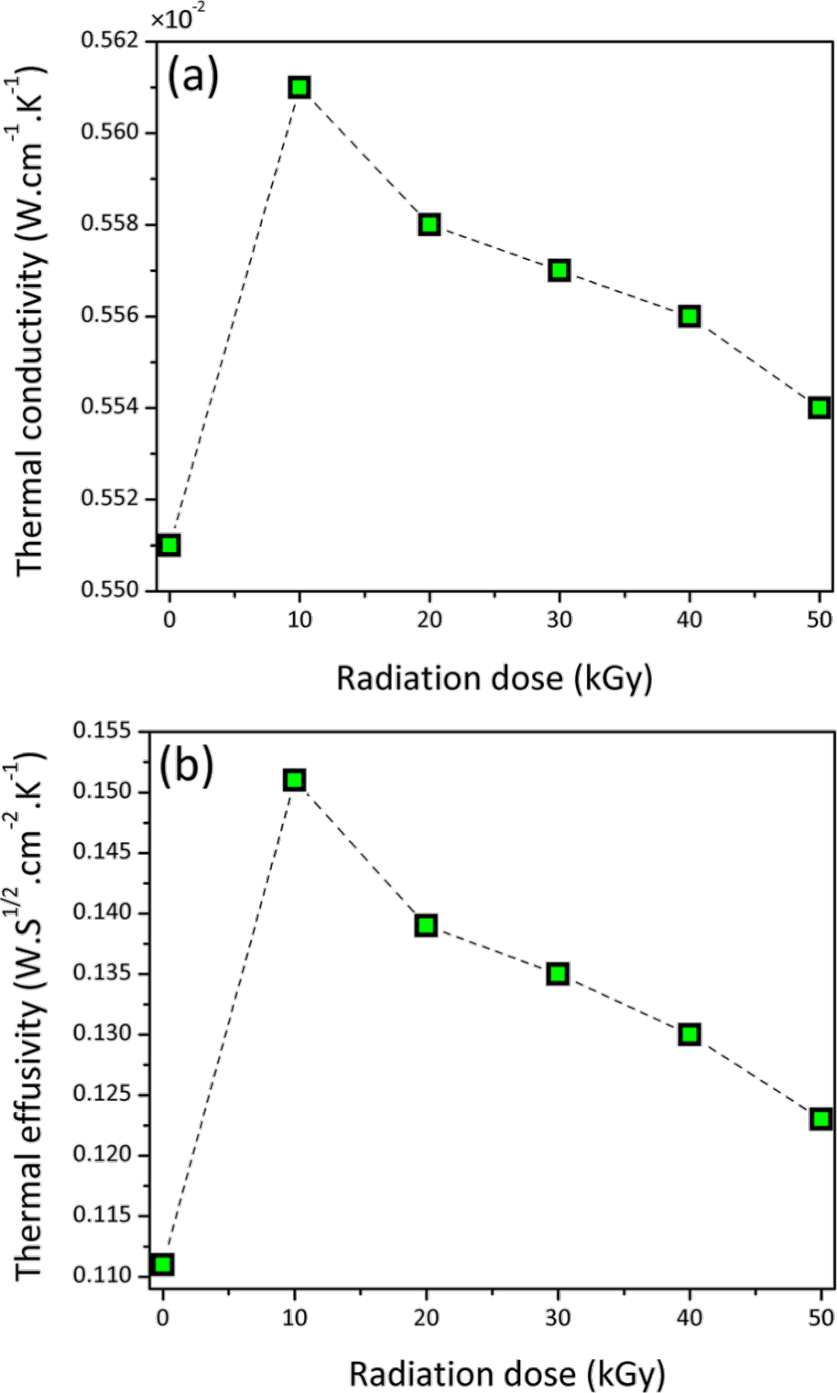
Trend of (a) thermal conductivity and (b) thermal effusivity of the PVA solution (0 kGy) and ZnS nanofluids as a function of the radiation dose.

The thermal conductivity of distilled water, PVA and ZnS nanofluids were measured and are listed in Table 2. The thermal conductivity of distilled water was measured to calibrate the set-up and agreed well with the reported value of the literature [29]. The thermal conductivity of the nanofluid was found to have a higher value than that of PVA due to the Brownian motion, convection, and heat diffusion in the presence of ZnS NPs [14]. The thermal conductivity of ZnS–PVA nanofluids decreases from lower to higher irradiation doses, as shown in Figure 7a. This decrement is explained by means of modification and breakdown in the chain structure of PVA upon gamma irradiation, as previously explained by FTIR results.

**Table 2:** Measured values of thermal conductivity (*k*) and thermal effusivity (*e*) in addition to the calculated values of thermal diffusivity (α) and specific heat (*C*_p_).

sample	*k* (W·cm^−1^·K^−1^) × 10^−2^	*e* (W·s^1/2^·cm^−2^·K^−1^)	α (cm^2^·s^−1^)	*C*_p_ (J·g^−1^·K^−1^)

distilled water	0.614	0.160	0.147	4.169
PVA solution	0.551	0.111	0.246	2.129
ZnS–PVA 10 kGy	0.561	0.151	0.138	3.870
ZnS–PVA 20 kGy	0.558	0.139	0.161	3.297
ZnS–PVA 30 kGy	0.557	0.135	0.170	3.116
ZnS–PVA 40 kGy	0.556	0.130	0.183	2.894
ZnS–PVA 50 kGy	0.554	0.123	0.203	2.600

According to [30], the principle of heat transport is explained by phonons where *k* can be written as follows [31]:

[8]
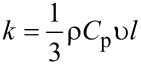


where ρ is the density, *C*_p_ is the specific heat at constant pressure, υ is the phonon velocity and *l* is the phonon mean free path. Therefore, changes in the phonon mean free path proportionally change the thermal conductivity.

**Figure 8:**
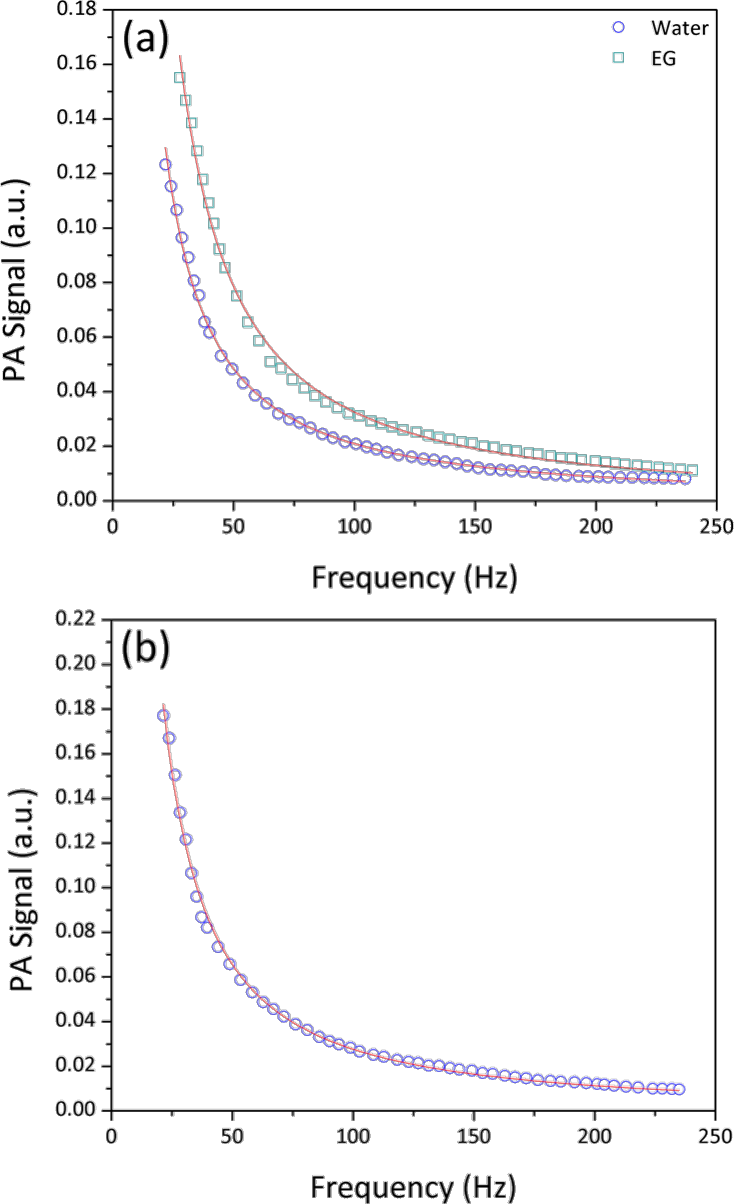
A typical PA signal as a modulation of frequency for (a) water and ethylene glycol, (b) ZnS–PVA nanofluids after irradiation with 50 kGy. The solid curve and hollow symbols indicate the theoretical fitting and experimental data, respectively.

Figure 8 shows the typical PA signal as a function of frequency for (a) distilled water and ethylene glycol and (b) ZnS–PVA at 50 kGy. In order to calibrate the set-up, the thermal effusivity of distilled water and ethylene glycol were measured and found to be 0.160 and 0.082 (W·s^1/2^·cm^−2^·K^−1^), which perfectly agreed with the reported values of the literature [32–34]. The thermal effusivity of distilled water, PVA and ZnS–PVA nanofluids are given in Table 2. The value of thermal effusivity decreased upon the increase of the radiation dose, as illustrated in Figure 7b. The decrement of thermal effusivity can be also explained by a decreasing chain length of PVA and, consequently, a shorter phonon mean free path due to the bond scission of PVA upon irradiation.

The *C*_p_ and α values of distilled water, PVA and ZnS–PVA nanofluids were then calculated through the relationships of *C*_p_, α, *e* and *k* [35]:

[9]
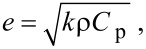


[10]
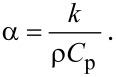


In order to measure the value of *C*_p_ and α, the mass of a given volume of each sample (35 mL) was determined, and so the density of the samples calculated. The calculated density of distilled water and the samples were found to be 1.00 and 1.05 g·cm^−3^, respectively. The *C*_p_ of samples was then calculated through Equation 9 by using formerly known values of *e*, *k* and ρ. Finally, Equation 10 was applied to calculate the value of α. The calculated values of *C*_p_ and α are also listed in Table 2. It was found that, in contrast to the values of *k*, *e* and *C*_p_, α increases with the irradiation dose, because it is inversely proportional to *C*_p_.

## Conclusion

The ZnS–PVA nanofluids were successfully synthesized by using gamma irradiation. The PVA was acetylated by residual acetate from the zinc acetate source, which leads to a higher viscosity of the samples. In addition, the chain structure of PVA was modified as a consequence of the high energy of gamma irradiation. The average size of the spherical ZnS NPs was found to increase from 53 to 59 nm with increasing doses from 10 to 50 kGy. The ZnS NPs exhibit zinc blende structure with the crystal lattice parameter of 5.4 Å and a cell volume of 157.46 Å^3^. The electronic absorption spectra revealed two absorption peaks located at 310 and 260 nm due to the ZnS NPs and carbonyl groups in the PVA structure, respectively. The band gap of ZnS NPs varied from 3.74 to 3.68 eV from lower to higher irradiation doses, which hints to a weak quantum confinement. Thermal conductivity and effusivity of the samples exhibited an increment upon the irradiation due to the chain scission of PVA. In contrast, the calculated value of thermal diffusivity was increased due to the decrement of specific heat since these two parameters are inversely proportional.

## Experimental

In this work, ZnS–PVA nanofluids were prepared by a room temperature radiolytic method using ^60^Co gamma irradiation. In a typical procedure, five different aqueous solutions of PVA (5 wt %) were prepared in a water bath for 1 h at 75 °C. Subsequently, the same amount, 10^−2^ molar ratio with respect to water, of Zn(CH_3_COO)_2_ and Na_2_S_2_O_3_ were added and the solution was stirred for 2 h. The solutions were finally irradiated with doses from 10 to 50 kGy.

The chemical structure of samples were investigated by Fourier transform infrared spectroscopy (Perkin Elmer model 1650), the morphology of samples was observed by transmission electron microscopy (JOEL 2010F UHR) operated at 200 kV, the structure of the final products was confirmed by powder X-ray diffraction in the 2θ range of 4 to 60° by using Philips X-ray diffractometer (7602 EA Almelo) with Cu Kα radiation (λ = 0.1542 nm). Optical absorption spectra were recorded by using a UV–vis spectrometer (Shimadzu-UV1650PC) in the wavelength range of 250 to 400 nm. The thermal conductivity and thermal effusivity of the samples were measured by transient hot wire (THW) and photoacoustic technique, respectively.

The thermal conductivity of the ZnS–PVA nanocomposites was measured by a Decagon devices KD2 Thermal analyzer where the transient line heat source is used to determine the thermal response. This device has 5% accuracy over the 5 to 40 °C temperature range. The device basically contains a single-needle sensor attached to a handheld readout unit. The sensor works as both heat source and thermometer. Technically, a single run experiment took 2 min. For the first 30 seconds, the probe was heated by a known current, and subsequently the device continued running for another 1.5 min to provide a steady temperature. The probe is also equipped with a thermistor that can detect and store the changes in temperature. Finally, the thermal conductivity was computed by the temperature difference versus time.

The thermal effusivity of samples was measured by a photo-acoustic technique based on Rosencwaig–Gersho theory [36]. The experimental and theoretical fitting of this method were comprehensively explained in our previous work [37].
